# Medical physics in developing countries: looking for a better world

**DOI:** 10.2349/biij.4.1.e29

**Published:** 2008-01-01

**Authors:** H Zaidi

**Affiliations:** Division of Nuclear Medicine, Geneva University Hospital, Geneva, Switzerland

**Keywords:** Medical physics, developing countries, education, training, international organisations

## Abstract

Medical physics has been identified as one of the key areas that need to be developed to improve healthcare. However, the level achieved in developing countries represents a stark contrast to the level that exists in Western Europe or North America. The challenge for developing countries is to build the required infrastructures, to acquire the equipment, to attract highly qualified professionals and to develop education and training programs and political policies for effective and accessible care within budgetary constraints. The state-of–the-art technological developments in medical physics cannot be viewed as a uniform reality all over the world. There is, of course, a wide difference in emphasis and approach when dealing with developing countries, compared to developed nations. As quality assurance and cost-benefit guidelines in the practice of radiation therapy and diagnostic imaging are being developed and debated in developed countries, the perspectives of the availability and standards of healthcare taken for granted in these countries stand in stark contrast to the level administered in developing countries. In this contribution, the overall situation of medical physics in developing countries and the barriers to improvement are discussed, and some possible solutions and ways to bridge the gap between developed and developing countries are suggested.

## INTRODUCTION

Medical physics can be one of the most challenging and rewarding applications of physics in society today [1, 2]. The historical development of diagnostic imaging and radiation therapy is marked by numerous significant scientific and technological accomplishments driven by an unprecedented interdisciplinary collaboration between physicists and physicians [3]. However, for many reasons, the state-of-the-art technological developments in medical physics cannot be viewed as a uniform reality all over the world. There is, of course, a wide difference in emphasis and approach when dealing with developing countries, compared to developed nations. As quality assurance and cost-benefit guidelines in the practice of radiotherapy, nuclear medicine and diagnostic radiology are being developed and debated in developed countries, the perspectives of the availability and standards of healthcare taken for granted in these countries stand in stark contrast to the level administered in developing countries. A typical example stressing this desolate disparity is the commercial introduction of dual-modality imaging systems and its widespread acceptance in clinical setting. Whereas cross-training requirements and guidelines for both the technologists who operate the combined units and the physicians (radiologists and nuclear medicine physicians) who interpret the images are being debated in developed nations [4, 5], the clinical relevance of this promising technology is still being questioned in many developing countries. Worse still, it is likely to take a while before clinicians will be prepared to adjust the way they used to practice medicine to take full advantage of novel state-of-the-art technologies available today. Moreover, despite the favourable economic situation and the availability of financial resources in some of these countries, it might take a long time until the decision makers realise the potential and cost-effectiveness of these novel technologies.

It is unfortunate to note that most developing countries still rely on developed countries to provide education and training programs whereas parts of the needed equipment is provided through international donation programs of used equipment, such as the one run by the International Organization for Medical Physics (IOMP). In recent years, medical physics organisations have begun to rely heavily on education and training in order to achieve and maintain the high standards required of today's healthcare providers. In addition, a number of non-profit organisations are still providing assistance to developing countries; but overall significant incremental improvement has yet to be achieved. Much has been written on this subject for awareness purposes and for expansion of guidance and assistance from international governmental and professional organisations working with national medical physics societies to improve medical physics on a country-by-country basis [6-11]. This paper discusses the current situation of medical physics in developing countries and the barriers to improvement, as well as examines the needs, in terms of equipment, education and training, in view of recently proposed guidelines.

## MEDICAL PHYSICS IN DEVELOPING COUNTRIES

Despite the remarkable progress achieved during the last decade in terms of acquisition of novel technologies, equipment and staffing, medical physics in developing countries is still well below the level required to provide adequate professional support to clinical diagnostic imaging and radiation therapy facilities available today. Africa is the least developed continent with respect to medical physics resources in terms of equipment and qualified professional staff, and requires particular attention. The basic needs in the delivery of healthcare are still profound in this part of the world. An analysis of available resources for medical physics support in these countries is urgently needed to serve as a baseline to plan future development [12]. This is indeed very hard to realise in practice, given the lack of reliable information and the rather obscure channels followed to plan and build new infrastructures and to acquire costly equipment in many of these countries.

A common facet shared by developing countries is that radiation protection procedures are not as heavily regulated as in developed countries and radiotherapy/diagnostic imaging facilities are less computerised. The lack of standard electronic patient record handling utilities available today in modern healthcare facilities is also worth emphasising given its negative impact on the daily management of patients and the availability of reliable statistics. Private facilities are limited and are, in general, prohibitively expensive for the average person in these economically depressed nations. The fraction of patients who receive indicated treatment is further reduced by social and economic factors and unfavoured individuals are denied services. Availability of radiation therapy and diagnostic imaging facilities is usually concentrated in the capital cities; besides, the local governments recognise the need to decentralise medical care. Many barriers, both practical and organisational, stand between the patient and necessary care. These problems of availability and needed implementation of strategies for cancer diagnosis and treatment are common in developing countries [6-10]. From my own experience, it would appear that the optimisation of current resources is more important than obtaining additional equipment and resources. Adequate education, training and application of standard diagnostic and therapeutic approaches and techniques should improve healthcare delivery.

The IOMP, through its statutes, encourages the worldwide establishment of professional medical physics societies and puts particular effort into stimulating their creation and growth in developing countries [13]. Among the objectives of national medical physics societies is to provide information and guidance on the training, responsibilities, organisational relationships, and roles of qualified staff in the field of medical physics. The major and most serious problem facing most of these societies is the absence of a professional status for medical physicists. This remains a major hurdle even in some countries from the developed world, following the refusal of the International Labour Organization (ILO) to recognise this profession (sequent to extensive lobbying by the IOMP) and to include it in the ILO classification of professions. In order to upgrade to such a status, it is necessary to make it a legal requirement that all radiation medicine facilities should employ medical physicists [13-16]. In its Malaga declaration, the European Federation of Organizations of Medical Physics (EFOMP) states that medical physics should be considered a ‘*regulated healthcare profession*’. Again this is far from being the rule even in developed nations, where the presence of a qualified medical physicist is legally required only in radiation therapy facilities.

One area of concern in most developing countries is that the number of qualified and experienced medical physicists per million of the population in the most important disciplines including radiotherapy, diagnostic radiology and nuclear medicine, is very low compared to levels achieved in Europe, for example. It has recently been realised in developed nations that the shortage of qualified medical physicists is a serious problem that requires particular attention [17-19]. The problem is catastrophic in developing countries, where some radiation therapy facilities and almost all diagnostic imaging units do not have access to expert physics support. The EFOMP survey [20] shows that the number of trained medical physicists (per million inhabitants) in Europe nowadays reaches 6 in radiation physics, 5 in nuclear medicine and 4 in diagnostic radiology. African countries present the lowest number of physicists per million of the population. This shortage requires very urgent attention by decision makers who should establish policies to limit the brain drain of qualified scientists or at least keep it to a reasonable level and to encourage the establishment of national medical physics societies to enable the growth of this specialty.

In a critical appraisal of the role of medical physicists in the developed world, Prof. S. Webb [2] advocated that “*medical physicists need to be actually doing medical physics, not spending too much time on administration, business, grant writing, scientific politics and unnecessary committee and professional society work*” … “*They should at the very least, stay active part-time research workers and/or clinical service scientists*.” This also applies to medical physicists practising in developing countries. However, the situation is always more complicated and the problems (including turf battles) faced in these regions of the world more difficult to handle. Medical physicists should be talented and have plenty of additional skills to be able to handle these issues efficiently. They should also be clever, diplomatic and excellent communicators to convince their administrators (usually inexperts in applications of physical sciences in medicine) about the importance of their work and its implications on healthcare delivery.

## IMPORTANCE OF EDUCATION AND TRAINING

Education and training are important factors for adopting, using, and supporting medical physics activities. Education of medical physicists should be tailored towards the requirements of healthcare institutions. It may not be suitable to implement educational programs in developing countries that are similar to those in developed countries. One of the arguments to support this view is that medical physicists in developing countries will not be involved in equipment design, in the way that their counterparts in developed countries are. Few medical physics programs have been initiated in developing countries. The educational programs usually comprise of general courses in physics and mathematics, and more specific courses in medical physics with special emphasis on radiation protection and radiotherapy physics. National medical physics societies should establish accredited education and training programs in their respective countries in collaboration with the IOMP, EFOMP, AFOMP or other regional societies [21]. If medical physicists would like to be considered professionals, they must have continuing educational requirements.

Appropriate education and training is the only way of preventing misuse and major accidents that are still occurring even in developed countries with advanced physics support [22]. Peer-review process between groups of medical physicists is another efficient way to allow taking preventive actions and to ensure collegial exchange of professional ideas and productive critique of daily clinical physics programs [23]. In addition, guidelines on how to report and deal with misconduct need to be developed and adopted by national medical physics societies [24].

The last decade has seen considerable paces taken towards the materialisation of professional training in medical physics throughout the world. The popularity of the Internet on a worldwide scale resulted in a huge amount of educational material available to students and trainees. Potential educational applications of these technologies in medical physics have been described elsewhere [25-27]. This has stimulated the emergence of many successful international collaborations aimed at establishing educational resources (electronic medical physics lectures and teaching files) and making them freely available for all users. However, the wide spectrum of contributions and the lack of coherent quality control has resulted in variable quality of available educational material. A noteworthy contribution supporting these initiatives came from two Leonardo European Union projects for European Medical Radiation Learning Development (EMERALD and EMIT), where a consortium of European universities and hospitals has developed training modules in medical radiation physics (x-ray diagnostic radiology, nuclear medicine, radiotherapy, ultrasound and magnetic resonance imaging) [28, 29]. Many other initiatives also reported on the development of educational material and other high-tech teaching tools and these efforts should be encouraged further as they are well received by young medical physicists from developing countries.

## ROLE OF INTERNATIONAL MEDICAL PHYSICS ORGANISATIONS

Existing international organisations involved in medical physics-related activities can be divided into two categories: governmental (IAEA, WHO) and non-governmental (IOMP, EFOMP, AFOMP, etc). Many efforts have been carried out by these organisations, particularly in developing countries, with the goal of improving the level of clinical physics support. These organisations have a noble role, although, in the author’s opinion, some approaches to assistance and training provided are not appropriately performed. To be effective, these organisations must be flexible and bring with them realistic training methods and technical support programs. On the other hand, many organisations benefit with tax advantages and circulating monetary resources, which include donations to developing countries. Even though this is not necessarily a drawback, it is important to identify additional motives for such organisations. Therefore, host countries should adopt the assistance that will best suit their own objectives. Acquiring donated equipment can be effective if it is carefully evaluated. Many donating parties do so for tax deduction purposes in their home countries and also to clear space occupied by unemployed equipment. Therefore donated equipment should only be accepted if it meets institutional needs. The IOMP programs for donation of used equipment and establishment of national medical physics libraries in developing countries are good examples of successful collaborative projects. The volunteers in charge of these projects, who are sacrificing their time, deserve to be congratulated for their efforts.

The main concerns raised about IAEA/WHO policies are that cooperation is conceived only with governmental organisations in member countries. The bottom line is that the decision-makers in some developing countries have limited knowledge about medical physics and lack the deep understanding of current international standards with respect to the requirements for establishing comprehensive cancer care and diagnostic imaging facilities with advanced physics support. For instance, it has been reported in many cases that some projects sponsored by the Agency are not appropriately managed. Those governments should be encouraged to consult the national medical physics societies and to seek their assistance. Due to lack of support by decision-makers, as well as often haphazard assistance programs that do not meet either short- or long-term objectives, many programs have been unsuccessful. Successful collaboration programs require the full support of decision-makers as well as national medical physics associations, in conjunction with appropriate planning.

## SUMMARY AND FUTURE PROSPECTS

The past century was the century of "big hit science" for medical physics where major discoveries and inventions were brought to the world by brilliant scientists that revolutionised the practice of medicine. Medical physics is not an undemanding and easy profession, and should never be considered as a ‘stomach job’ used to earn its living, but instead as a passion that should be given the place it deserves in our lives. We all recall the words of late Prof. Abdus Salam (1979 Nobel Laureate in Physics), who said that *scientists are very happy people because their job is also their hobby*. It goes without saying that this is the example that physicists from developing countries should follow. Novel technologies are driving the growth of healthcare delivery and cutting-edge biomedical research. Yet developing countries will not likely have a chance to access these technologies in the near future. Contrary to the opinions claiming that it is the duty of developed governments to translate and adapt those novel techniques to the particular needs of developing countries [2], I strongly believe that it is the role of the countries needing these technologies to acquire the knowledge required for translation and adaptation, as this task is not the priority for developed nations, where young medical physicists practicing in these countries are focused on building their careers.

Government officials can play a very important role by establishing effective long-term policy goals for physics support in healthcare institutions. Issues such as providing an adequate budget for training support and proper equipment can have a profound impact on healthcare services. Staffing levels may not depend on bed size, as compared to developed nations, since the average hospital in those countries has much more equipment compared to a hospital of the same size in a developing country. In many developing countries, the first need is to provide high quality physics support in clinical routine. National medical physics societies that already exist can play an important role in creating awareness and proper communications with higher authorities. Organising lectures, support to users, establishing policies and guidelines, all while assuring good productivity and motivation through objective methods, can have a positive impact. In view of the actual situation, there are still some political lessons to be learned. One important issue that needs to be stressed is the inclusion of national scientific non-governmental organisations in cooperation programs to control the efficient use of money and equipment. Education and training requirements for medical physicists should be harmonised around the world. This can be achieved only if local governments and national organisations adhere to such a project. Scientific information should not be “owned” but be freely available through the usual channels. Continuing efforts are needed to bridge the gap between developing and developed countries. In this regard, establishment of guidelines for proper medical physics education and clinical support in developing countries are urgently needed.
